# Managing a Colonoscopic Perforation in a Patient with No Abdominal Wall

**DOI:** 10.1155/2018/7175381

**Published:** 2018-07-10

**Authors:** Jayan George, Michael Peirson, Samuel Birks, Paul Skinner

**Affiliations:** Department of General Surgery, Northern General Hospital, Herries Road, Sheffield S5 7AU, UK

## Abstract

We describe the case of a 37-year-old gentleman with Crohn's disease and a complex surgical history including a giant incisional hernia with no abdominal wall. He presented on a Sunday to the general surgical on-call with a four-day history of generalised abdominal pain, nausea, and decreased stoma output following colonoscopy. After CT imaging, he was diagnosed with a large colonic perforation. Initially, he was worked up for theatre but following early senior input, a conservative approach with antibiotics was adopted. The patient improved significantly and is currently awaiting plastic surgery input for the management of his abdominal wall defect.

## 1. Introduction

Crohn's disease is a condition affecting approximately 145/100,000 people [1]. Within this population, 70% will undergo surgery and of that number 30–70% will require repeat or multiple procedures[2]. The management of these patients can be quite complex. Surgical intervention is normally reserved for when medical therapy has failed [3].

Colonoscopy is a common procedure performed for diagnostic and therapeutic purposes. It is estimated that up to fifteen million colonoscopies were performed in the United States in 2012 [4, 5]. Colonoscopic perforation is a serious complication and can occur at rates ranging from 0.016 to 0.8% [6–11].

We describe the case of a patient with a background of Crohn's disease resulting in multiple operations leading to loss of his abdominal wall (and a subsequent giant incisional hernia) presenting with colonic perforation. The patient presented on a Sunday to the general surgical on-call. Experienced registrars were present and the patient could have been taken to theatre. This case is unique, and many experienced clinicians are not likely to have encountered an abdomen such as this. To our knowledge, this is the first case of its kind.

## 2. Case Report

We report the case of a 37-year-old gentleman who presented on a Sunday to the general surgical on-call with a four-day history of generalised abdominal pain postcolonoscopy. He had associated nausea and slightly reduced stoma output.

Past medical history includes asthma and Crohn's disease which had settled at the time leading up to the colonoscopy. There were no known drug allergies, and the patient takes azathioprine, salbutamol, and beclometasone. He is a nonsmoker and drinks minimal alcohol.

Past surgical history includes a complicated appendicectomy in 2007 resulting in a colostomy; a colonic perforation and retroperitoneal abscess secondary to Crohn's disease led to an ileostomy in 2010, and the ileostomy was reversed with an ileocolonic anastomosis formed in 2012. Anastomotic dehiscence occurred leading to major sepsis with abdominal wall breakdown and abdominal compartment syndrome. A debridement of the area was performed and left as a laparostomy, and an ileostomy was reformed. The area was later covered by a large skin graft in 2012. His colonoscopy was part of a preoperative workup for a procedure in a quaternary centre to assess his viability to repair his complex hernia.

**Figure 1:**
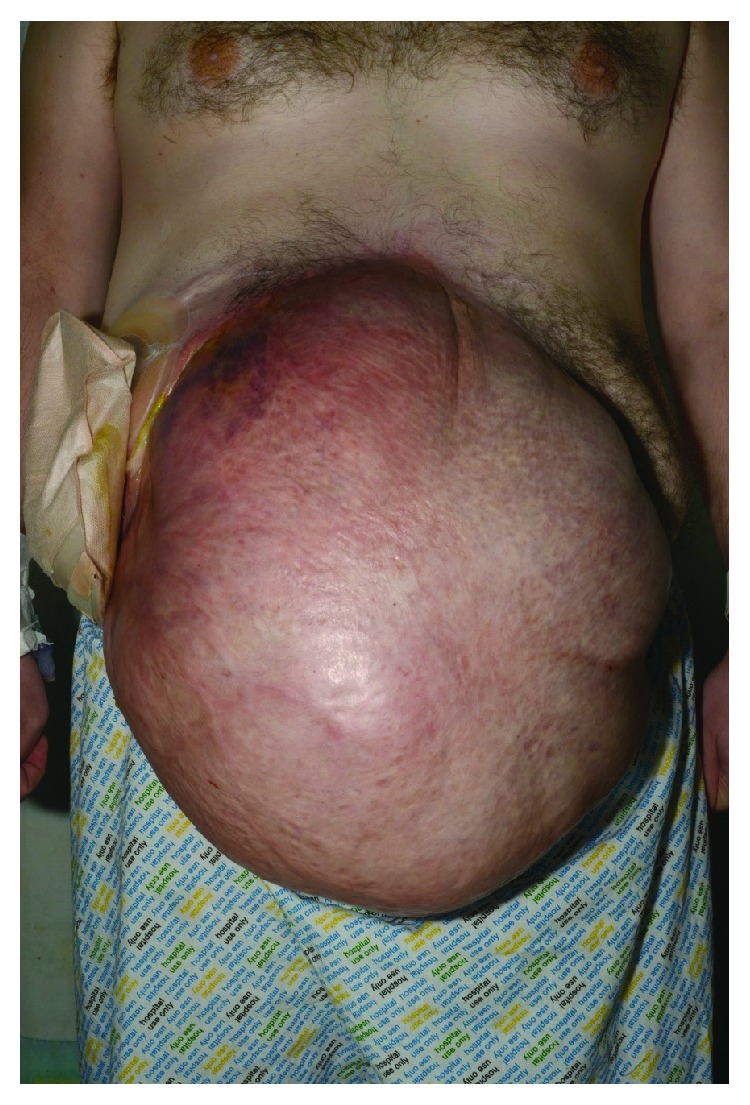
Anterior view of large abdominal hernia.

**Figure 2:**
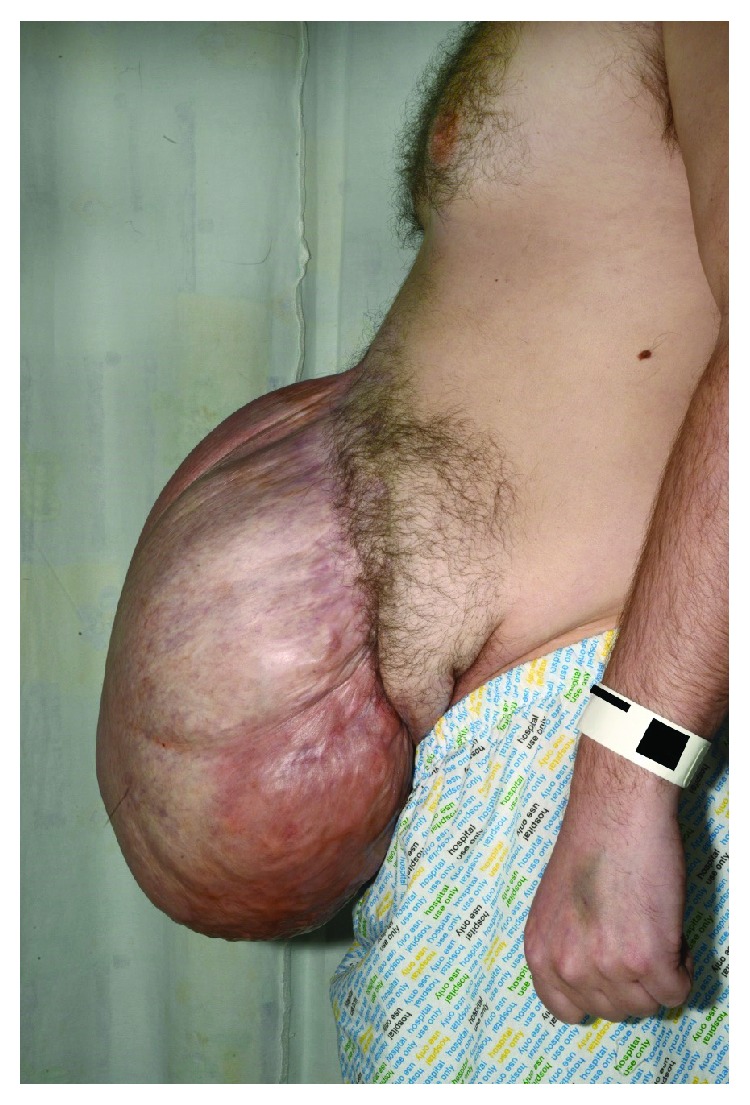
Lateral view of large abdominal hernia.

**Figure 3:**
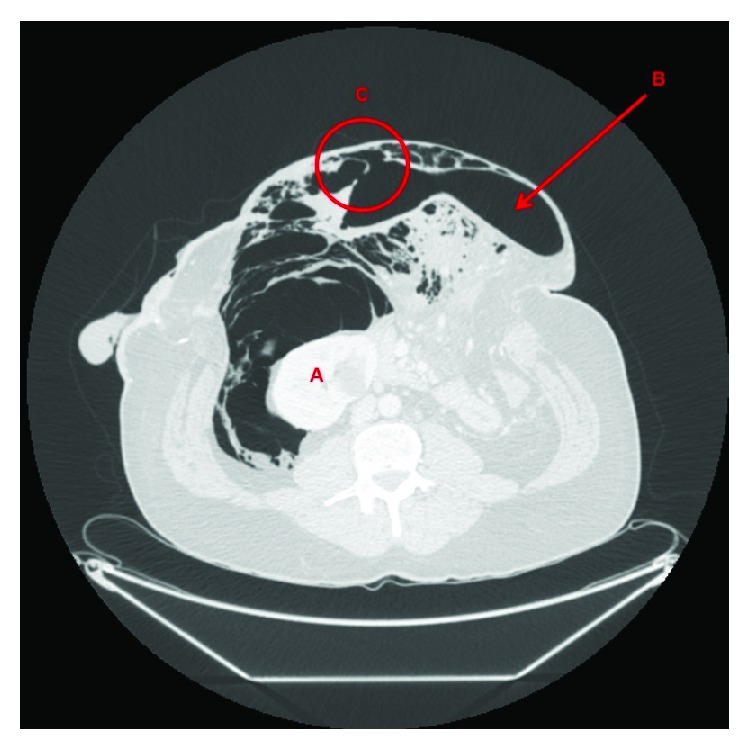
Axial CT image showing (A) retroperitoneal free gas around the right kidney (not typical with transverse colon perforation), (B) transverse colon, and (C) perforation of the transverse colon.

On examination, his heart rate was 117 beats per minute (bpm), blood pressure 128/81 mmHg, respiratory rate 15, and oxygen saturation 98% on air. There was a large mass overlying the hernia to the left of the midline and on abdominal palpation; the mass was ballotable with crepitus, was slightly tender, and had a cough impulse (Figures 1–3). In addition, a stoma was present. The chest was clear to auscultation, and GCS was 15/15.

Bloods on admission revealed a C-reactive protein of 219 mg/L (0–5 mg/L) and were otherwise unremarkable.

### 2.1. Computed Tomography Scan of the Abdomen and Pelvis with Contrast (Day of Admission)

**Figure 4:**
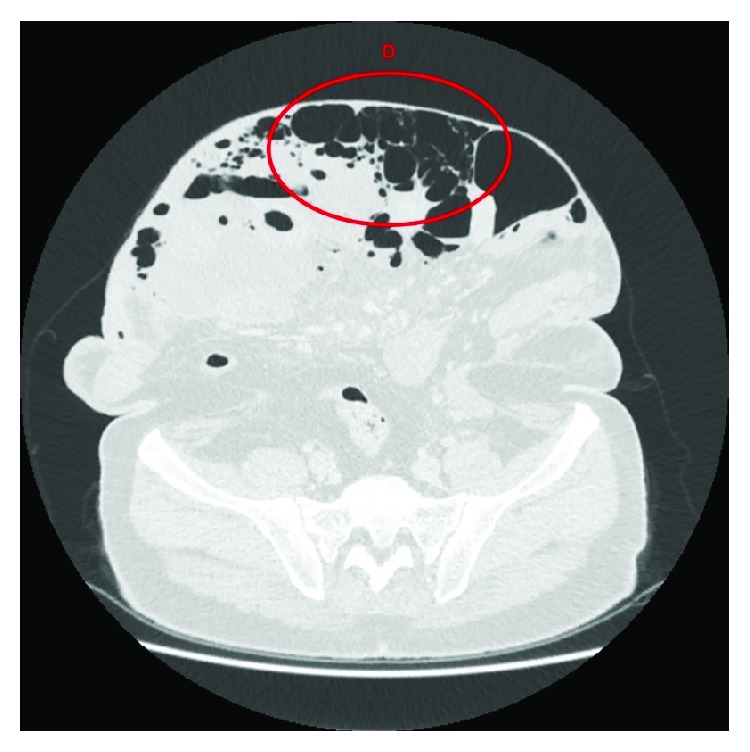
Axial CT image showing (D) septated free gas, presumed to be located in the mesentery of the colon.

There is a huge amount of free air, which is most likely secondary to a recent colonoscopy that has probably blown off the ascending colon stump. The colon cannot be traced beyond the midtransverse colon in the current scan (Figure 4). A large midline abdominal wall hernia containing several bowel loops with most of the gas seeping into the mesentery within the hernia can be seen. Part of the gas is also seen in the intrahepatic and right perinephric space.

The patient was managed with an ABCDE approach. Tazobactam with piperacillin (Tazocin) was administered as per local guidelines. Intravenous fluids, analgesia, and monitoring of output were commenced.

Given the result of the computed tomography scan, the surgical registrars had consented the patient for a laparotomy plus proceed as necessary. This was halted when the consultant on-call reviewed the patient an hour later.

A conservative approach was adopted, and the patient was discharged just days following admission. He did very well and is currently undergoing review at one of our quaternary centres for his abdominal wall reconstruction.

Following his admission and conservative management, contact was made with the quaternary centre and they reported that the colonoscopy had gone to plan and was carried out meticulously and there was no evidence of a perforation; it was noted that there was evidence of inflammation macroscopically and microscopically and this was determined to be nonspecific.

## 3. Discussion

Colonic perforations are a rare complication, but when they occur, they can be quite significant with morbidity rates of up to 55% [12]. Mortality can range from 0 to 20% [13, 14]. A systematic review examining imaging techniques to diagnose colonic perforation suggests CT in the first instance unless the patient is haemodynamically unstable, where a decubitus or upright abdominal film could be used instead [6]. Our patient was haemodynamically stable and was referred to us having had a CT scan confirming perforation, and prompt action was taken (Figures 3 and 4).

A recent systematic review indicated that the management of the stable colonic perforation is to observe and treat conservatively unless any of the following findings are present: diffuse peritonitis on examination, heart rate > 100 bpm, temperature > 38°C or <36°C, respiratory rate > 20 breaths per minute, PaCO2 < 2.7 kPa, white blood cell count > 12 × 10^9^/L or <4 × 10^9^/L or >10% immature (band) forms, mean arterial pressure < 65 mmHg or relative hypotension, or altered mental status [6]. Our patient was haemodynamically stable with raised inflammatory markers and a very pronounced abdominal hernia; given the fact that he had little abdominal wall, he would not present as a typical peritonitic abdomen (Figures 1 and 2). This led to the concern that he needed to go to theatre and the patient was consented and prepped; however, he was clinically stable and following senior input, conservative management continued.

Very few studies have looked at which antibiotic is appropriate, but aerobic and anaerobic cover is suggested [15]. We used Tazocin as per our local guidance for intra-abdominal sepsis, and this has good cover for both types of organisms.

Hawkins et al. have developed an algorithm based on best evidence to manage the patient surgically: for a small perforation, they advocate that surgical repair is preferable but for large perforations, obstruction, malignancy, or inflammatory bowel disease, a surgical resection is more appropriate [6]. In this case, surgery for the patient would be incredibly difficult and would carry a significant risk given his lack of an abdominal wall, especially during postoperative recovery.

The biggest determining factor in this patient's management was early consultant input. Whilst the patient was worked up for theatre, the consultant on-call was informed, and this prompted an early senior review which led to the conservative management approach being followed. This is a reminder for all trainees to focus on the pathology and the patient's clinical status in initiating treatment and not to be distracted by the obvious abdominal wall defect and CT images (Figures 1–4).

## References

[1] Rubin G. P., Hungin A. P., Kelly P. J., Ling J. (2000). Inflammatory bowel disease: epidemiology and management in an English general practice population. *Alimentary Pharmacology and Therapeutics*.

[2] Duepree H.-J., Senagore A. J., Delaney C. P., Brady K. M., Fazio V. W. (2002). Advantages of laparoscopic resection for ileocecal Crohn’s disease. *Diseases of the Colon & Rectum*.

[3] Patil S. A., Cross R. K. (2017). Medical versus surgical management of penetrating Crohn’s disease: the current situation and future perspectives. *Expert Review of Gastroenterology & Hepatology*.

[4] Jaberoo M.-C., Joseph J., Korgaonkar G., Mylvaganam K., Adams B., Keene M. (2013). Medico-legal and ethical aspects of nasal fractures secondary to assault: do we owe a duty of care to advise patients to have a facial x-ray?. *Journal of Medical Ethics*.

[5] Joseph D. A., Meester R. G. S., Zauber A. G. (2016). Colorectal cancer screening: estimated future colonoscopy need and current volume and capacity. *Cancer*.

[6] Hawkins A. T., Sharp K. W., Ford M. M., Muldoon R. L., Hopkins M. B., Geiger T. M. (2018). Management of colonoscopic perforations: a systematic review. *The American Journal of Surgery*.

[7] Kim J. S., Kim B.-W., Kim J. I. (2013). Endoscopic clip closure versus surgery for the treatment of iatrogenic colon perforations developed during diagnostic colonoscopy: a review of 115,285 patients. *Surgical Endoscopy*.

[8] Lohsiriwat V. (2010). Colonoscopic perforation: incidence, risk factors, management and outcome. *World Journal of Gastroenterology*.

[9] Silva A. C., Pimenta M., Guimaraes L. S. (2009). Small bowel obstruction: what to look for. *Radio Graphics*.

[10] Scott J. W., Olufajo O. A., Brat G. A. (2016). Use of national burden to define operative emergency general surgery. *JAMA Surgery*.

[11] Magdeburg R., Collet P., Post S., Kaehler G. (2008). Endoclipping of iatrogenic colonic perforation to avoid surgery. *Surgical Endoscopy*.

[12] Tam M. S., Abbas M. A. (2013). Perforation following colorectal endoscopy: what happens beyond the endoscopy suite?. *The Permanente Journal*.

[13] Kim H.-H., Kye B.-H., Kim H.-J., Cho H. M. (2014). Prompt management is most important for colonic perforation after colonoscopy. *Annals of Coloproctology*.

[14] Teoh A. Y. B., Poon C. M., Lee J. F. Y. (2009). Outcomes and predictors of mortality and stoma formation in surgical management of colonoscopic perforations: a multicenter review. *Archives of Surgery*.

[15] Magdeburg R., Sold M., Post S., Kaehler G. (2013). Differences in the endoscopic closure of colonic perforation due to diagnostic or therapeutic colonoscopy. *Scandinavian Journal of Gastroenterology*.

